# Assessment of Automated Analyses of Cell Migration on Flat and Nanostructured Surfaces

**DOI:** 10.5936/csbj.201207004

**Published:** 2012-11-21

**Authors:** Cristian Grădinaru, Joanna M. Łopacińska, Johannes Huth, Hans A. Kestler, Henrik Flyvbjerg, Kristian Mølhave

**Affiliations:** a Department of Micro- and Nanotechnology, Technical University of Denmark, Kgs. Lyngby, Denmark; b Neural Information Processing, University of Ulm, Ulm, Germany; c Department of Gastroenterology and Endocrinology, University Hospital of Marburg, Marburg, Germany; d Internal Medicine I – Gastroenterology, University Hospital Ulm, Ulm, Germany

**Keywords:** Time lapse microscopy, cell tracking, software, cell motility, cell migration, image analysis, nanotechnology

## Abstract

Motility studies of cells often rely on computer software that analyzes time-lapse recorded movies and establishes cell trajectories fully automatically. This raises the question of reproducibility of results, since different programs could yield significantly different results of such automated analysis. The fact that the segmentation routines of such programs are often challenged by nanostructured surfaces makes the question more pertinent. Here we illustrate how it is possible to track cells on bright field microscopy images with image analysis routines implemented in an open-source cell tracking program, PACT (Program for Automated Cell Tracking). We compare the automated motility analysis of three cell tracking programs, PACT, Autozell, and TLA, using the same movies as input for all three programs. We find that different programs track overlapping, but different subsets of cells due to different segmentation methods. Unfortunately, population averages based on such different cell populations, differ significantly in some cases. Thus, results obtained with one software package are not necessarily reproducible by other software.

## Introduction

Cell migration plays an essential role in many biological processes: e.g. wound healing, embryogenesis, inflammation, and metastasis where uncontrolled cell migration can lead to tumor spreading and hence can cause cancer progression. Studying these processes frequently require cell tracking, and most motility studies of monolayer cultures involve fluorescent labeling of cells, which allows for fluorescence microscopy studies [1][2]. This technique either requires extensive mutagenesis to have fluorescent protein expressed by the cell type under study, or is limited by the fact that membrane-attached or permeable fluorescent drugs often alter cell behavior [3]. In sparse cultures, cells can have sufficiently good contrast against the background that their boundaries can be identified with bright field microscopy without labeling [4a][4b]. This can be done manually with point-and-click methods at great expense of time and labor [5][6][7][8][9][10]. When cells in only a few images have to be tracked, this is not a challenge. However, when long time-lapse sequences of motile cells need to be analyzed, this approach is impractical, raising the need for a reliable automated cell tracking program and assessment of the robustness of the method of analysis.

Representative programs for sparse cell culture analysis are listed in Table 1. The majority of such programs (not included in Table 1) are designed for tracking fluorescently labeled cells [1][2] [15][16][17][18]. The programs designed for use with light microscopy images track either the cell nucleus [19][20] or the entire cell [21][11][22][12]. The cell position can be defined as: (i) the center of the nucleus [6]; (ii) the centroid of the cell's perimeter as seen in the light microscope [23]; (iii) the centroid of the cell's footprint as seen in the light microscope [4]; and (iv) the centroid of the actin cytoskeleton of fluorescently labeled cells [24]. Most of these programs are not open source, and may be difficult to adapt to the specific purposes of a given experiment. In other cases the complexity of the mathematical procedures used for boundary identification may be a hurdle to adapting the code to a specific purpose [2][20][17]).

**Table 1:** Overview of representative programs for cell tracking by time-lapse light microscopy.

Program	Publisher/Seller	Commercial	Open source	Coordinate output format
Volocity	Improvision	Yes	No	Text
Imaris	Bitplane	Yes	No	Excel
Autozell [11]	Universität Bremen	Yes	No	Text
TLA [12]	University of Ulm	No	Yes	Excel/Text
CellTrack [13]	Ohio State University	No	Yes	Text
ImageJ plugins [14]	ETH & UCSF	No	Yes	Text

There are two main approaches to cell tracking in the current state-of-the-art [25][21][16][20][26][18]. One approach is frame-by-frame image segmentation and tracking [15][27]. In the first step, the object candidates are detected in a given frame on the basis of their specific properties (border, texture, color). This approach is efficient when object borders are sharp, and it is commonly used with fluorescently labeled cells and other high-contrast images. The other approach consists in optimizing a parameterized model shape to fit the model to the cells in a frame. Instead of tracking all objects in the frame, this method focuses on those candidates which correspond to the chosen model shape [28][29]. As with the first approach, detected objects are paired between consecutive frames in order to produce tracks.

In order to address the issue of background removal, and to compare the performance of different programs on real data against a baseline which we fully understand and control, we here introduce a new cell tracking program, which we call PACT (rogram for Āutomated ell racking). PACT is suitable for tracking motile cells on flat and nanostructured surfaces, and is simple enough for users to freely modify it according to their experimental needs. Since nanostructured surfaces are currently of great interest as cell culture substrates and can appear as a highly non-uniform background in the image, we emphasize the use of a reliable spatial image filter here – see details in the materials and methods section, and SuppInfo.zip file for the Matlab code.

Test results are presented for the performance of PACT, which is also compared to the performances of other programs (*TLA* [12] and *Autozell* [11]): efficiency with regard to object detection, accuracy of centroid positioning, and segmentation performance in the context of time-lapse analysis. A statistical analysis is then performed on the ensemble of individual tracks of cells to determine the overall cell population motility statistics in a movie of NIH 3T3 fibroblasts on glass. From the tracks, the auto-covariance function of the velocity is estimated (Equation S1 in supp. info.). It is well described by a simple exponential function with characteristic time, *P*, the *persistence* *time* of the motility (data shown in supp. info sect. 4). We also determine the amplitude of the velocity auto-covariance function, φ_0_, which is approximately equal to the mean squared velocity of the cells. Results of this analysis obtained with PACT, TLA and Autozell are compared.

Despite the need for cell tracking programs and algorithms, we found few studies evaluating their performance [11][12][26]. To our knowledge, this paper is the first comparative analysis of tracking programs with the scope of providing reliable data acquisition routines for developing motility models. We find considerable sensitivity of results to the specific algorithm/software employed. Thus, the tracking algorithm and its effect on results should be well documented in future studies, for instance as described in this study.

## Materials and Methods

### Cell Culture and Substrate Nanofabrication

We have imaged HeLa and NIH 3T3 cells on a microscope glass slide (Thermo Scientific, Menzel-Gläser), and NIH 3T3 cells on flat silicon and silicon black [4b]. Silicon black samples were fabricated by reactive ion etching of silicon substrates [30][31].

The cell lines were obtained from Risø National Laboratory, Denmark. The cells were grown in Dulbecco's Modified Eagle Medium: Nutrient Mixture F-12 (DMEM/F12) + GlutaMAX (Invitrogen) supplemented with either 10% fetal bovine serum – FBS (Sigma) in the case of HeLa cells, or 10% of newborn calf serum for NIH3T3 cells, 2 mM L-glutamine (Sigma), 100 U/mL penicillin (Sigma), 100 µg/mL streptomycin (Sigma) and grown until confluency. Cells were harvested by a standard trypsinization method, then seeded at a concentration of 5 x 10^4^ cells per each well of 24-well plate and cultured on the tested materials (1cmx1cm) for 1 or 3 days. The time-lapse microscopy experiments were performed in a home-made cell culture chamber equipped with a bubble trap and adjustable medium flow, mounted on a temperature-controlled microscope stage.

For the fluorescence microscopy experiments performed to compare brightfield images with fluorescence microscopy images of the actin cytoskeleton of the cells, the cells were treated with 2% glutaraldehyde in 0.05M cacodylate buffer for 15-20 minutes at the room temperature, washed in 1xPBS containing 0.05% Tween-20, and permeabilized with 0.1% Triton X-100 in 1xPBS for 1-5 minutes at room temperature. After being washed three times with 1xPBS containing 0.05% Tween-20, the cells were incubated in TRITC-conjugated phalloidin (Sigma-Aldrich) for 30 minutes, and rinsed three times with 1xPBS containing 0.05% Tween-20.

### Data and Image Sequence Acquisition

The videos contain 8-bit grayscale images recorded with a temporal resolution of 2-10 minutes. Each image pixel has a size of 0.977 x 0.977 µm and the resolution of the images was 1024 x 768. The recording device was a Zeiss Axiotech microscope equipped with a 10x Zeiss objective with a 19 mm working distance and a field of view of 1000 x 750 µm^2^ and a Labview-controlled microscope stage. The acquisition technique was bright field microscopy, and reflected light microscopy was used as many of the substrates were not transparent to light. The fluorescence and bright field microscopy experiments with fixed cells were performed on an Olympus BX51 upright microscope. Analysis of bright field images was performed using the TLA setup file provided by its authors. It may be found in the SuppData.zip file. By default, TLA tracks cells imaged in bright field microscopy by first applying a low-pass Gaussian filter of size 25x25 pixels and standard deviation 11 pixels. A Wiener low-pass filter in a 15x15 pixel mask around each pixel is applied to remove the pixel noise in the image, and this is followed by the actual segmentation process.

### PACT Program Implementation and Functionality

PACT was implemented using MATLAB (ver. 2008a) and is a text-based, interactive, open source application for the analysis of cell motility data with methods described by Selmeczi et al. [6]. This analysis requires accurate measurements of cell centroid coordinates throughout the duration of their observation.

*Processing* a time lapse movie in PACT consists of the following workflow: *Tracking* the individual moving objects through a sequence of images, where the objects have been localized by an image segmentation routine on images after a suitable image filtering process for optimal segmentation results. PACT employs a combination of two of the simplest and fastest segmentation methods: thresholding and edge detection. The result of a PACT processing is a time-ordered list of centroid coordinates for each object that is tracked. Root mean square deviation filtering (*RMSD-filtering*, see point (v) below) is an optional last step of processing and removes tracks of objects whose displacement is below a user-defined threshold. This option automatically removes tracks of non-motile cells, such as cells immobilized on the surface and lysed cells.

*Post-processing* is the manual screening of the processed data using criteria of biological relevance, which results in final identification of biologically relevant *cells* amongst the objects tracked. Human judgment is needed to decide which tracks are of sufficient quality for inclusion in the data analysis. This involves discarding segments of tracks where cells either: (a) undergo division; (b) come into contact with one another; (c) carry foreign objects; (d) and optionally cells that do not display normal motility patterns (i.e. those that do not extend lamellipodia or filopodia).

The source code of PACT is provided in Supporting Information 2. The program takes the recorded movie frames as input and outputs the cell centroid coordinates. The frames must be located in the same folder as the source code of the program and a new folder ("processed") is created where overlay images of original frames and the contours and cell centroids are stored. The section “*working parameters*” in the main program may be used to change the name of the input files and to invert the images if needed. The frame may be color or gray-scale; PACT will remove the color component if present. 8-bit depth is sufficient if the contrast is good, else 16-, 24-, etc. may be needed.

For image filtering, a band-pass filter that is part of ‘IDL (Interactive Data Language) Particle Tracking’ package [32] is first applied to remove pixel noise and non-uniform background illumination effects (Figure S1). This is done by convolving (○ operator) the image array *I* in two steps with a Gaussian function *G* and a boxcar function *B* and subtracting the result:

$$\mathrm{Filtered}I=(I^{T}{oG}^{T}){}^{T}{oG}^{T}-(I^{T}{oB}^{T}){}^{T}{oB}^{T}$$

In Fourier space this amounts to applying a radially symmetric mask function which features a Gaussian decline at high frequencies and an abrupt fall at low frequencies. The limits of this band-pass filter are also set up in the *working parameters* section: the high-frequency limit by the standard deviation of *G*, and the low-frequency limit by the width of *B*. We found that a high-frequency limit of 2-3 pixels works well for most flat surfaces, and that as much as eight pixels may be required for the highly noisy backgrounds that we have encountered when imaging cells on silicon black. We typically set the low frequency limit to 30-50 pixels as this corresponds approximately to the size of an average cell in our microscopy setup and efficiently filters inhomogeneous background illumination.

For the segmentation, PACT has a text-based, interactive user interface, which iteratively requests two parameters until the user is satisfied with the selection. The first parameter is the *peak exclusion threshold* which defines the minimal brightness of a spot in the band-pass filtered image required for inclusion on the object list. The peak (brightest pixel) of the object is marked as a red cross on the image. The cell contour shown as a blue line in the image is defined as the isohypse located at a user-defined percentage (*the contour cutoff*) of the peak level (See supp. info. Fig S1). The segmentation is done manually for the first and last frames in the movie, and all intervening frames are processed automatically with an average of the contour cutoffs and linear interpolation of the peak exclusion thresholds set for these two frames. The linear interpolation is especially useful for long movies where the background and/or object brightness may change slowly in time due to factors external to the experiment (see section (ii) below).

Further selection includes restrictions on minimal and maximal cell area (to remove unwanted objects such as small pieces of dust or large air bubbles and cell conglomerates from the analysis) and minimal distance between cells to ensure that only independent cells are detected. These parameters may also be set in the *working parameters* section.

The tracking process entails sorting this independent list of coordinates (determined at discrete times as the centroid of the cell footprint) into particle tracks using a routine part of ‘IDL Particle Tracking’ package [32]. If desired, it is possible to add a last exclusion criterion for removing the non-moving cells on the basis of an RMSD filter (see RMSD-filtering section (v) below.

### Assessment of Reliability

To assess the reliability of automated tracking and the relative performance of PACT to some other programs available, we have first evaluated the cell segmentation performance at the single-frame level. To that end we have done the following:

- Tested the segmentation efficiency of our code, namely how well it locates individual cells in comparison to the other programs available.
- Assessed how changes in focus can affect the accuracy of cell centroid positioning
- Compared PACT’s accuracy of centroid positioning in brightfield imaging and fluorescence imaging
- Evaluated the background removal efficiency on nanostructured surfaces by comparing segmentation and centroid position results from bright-field and fluorescence microscopy of the same sample.

We have also tested the performance of PACT and other programs at tracking motile cells by performing a statistical analysis of tracks:

- We study the effect of the RMSD-filtering procedure used to remove non-motile cells and its effect on the cell motility parameters.
- We compare pair-wise the statistics obtained by tracking with different programs.
- We compare the positional noise as function of software.
- We assess the reproducibility of our method by comparing cell track statistics determined from different movies of the same cell type/substrate-pair with PACT.
- We compare the velocity autocovariance functions calculated from tracks obtained with different programs

Below, these points are elaborated, and the ensuing discussion section deals with how these observations relate to each other and lead to the conclusions.

## Results

### (i) Segmentation efficiency

We tested segmentation on a movie frame of HeLa cells on a glass substrate, NIH 3T3 on a flat silicon substrate, and NIH 3T3 on a silicon black substrate with PACT, TimeLapseAnalyzer (TLA)[12] Autozell[11], and CellTrack[13]. A comparison of the number of objects identified by each program vs. the actual number, as determined by manual counting i.e. segmentation efficiency, is shown in Table 2.

**Table 2:** Number of objects segmented by the various cell tracking programs in bright field images (see the SuppInfo.zip file “HeLa-glass.tif” for HeLa/glass, “Si BF.jpg” for 3T3/Si, “Si black BF.jpg” for 3T3/Si black), before manual post-processing

	PACT	Autozell	TLA	CellTrack	Manual count
HeLa/glass	199	124	197	157	206
NIH 3T3/flat Si	107^1^	104	132^2^	160^1^	158
NIH 3T3/Si black	40	0	75^3^	0	48

1 The cell count for NIH 3T3/flat Si by PACT is underestimated due to this data being cumulated from five different images, as the band -pass filter of PACT removes the edge of the image prior to processing. This effect makes the cells on the edge inaccessible to segmentation. This is less obvious in the case of the HeLa/glass or NIH 3T3/Si black samples where the count was performed on a single image.

2 TLA occasionally assigns multiple centroids to one cell, and we have counted such instances as just one object in Table 2.

3 Wiener-filter noise-parameter lowered to 0.03 from the default of 0.05 for non-uniform background removal.

PACT has a cell segmentation efficiency comparable to that of TLA, and both are better than Autozell and CellTrack for HeLa/glass, while CellTrack performs better for 3T3/Si. The cell selection efficiency is very dependent on the software used and on the settings used in the segmentation. With training in how to use software, users can achieve 80-90% count rates compared to manual counting.

**Figure 1:**
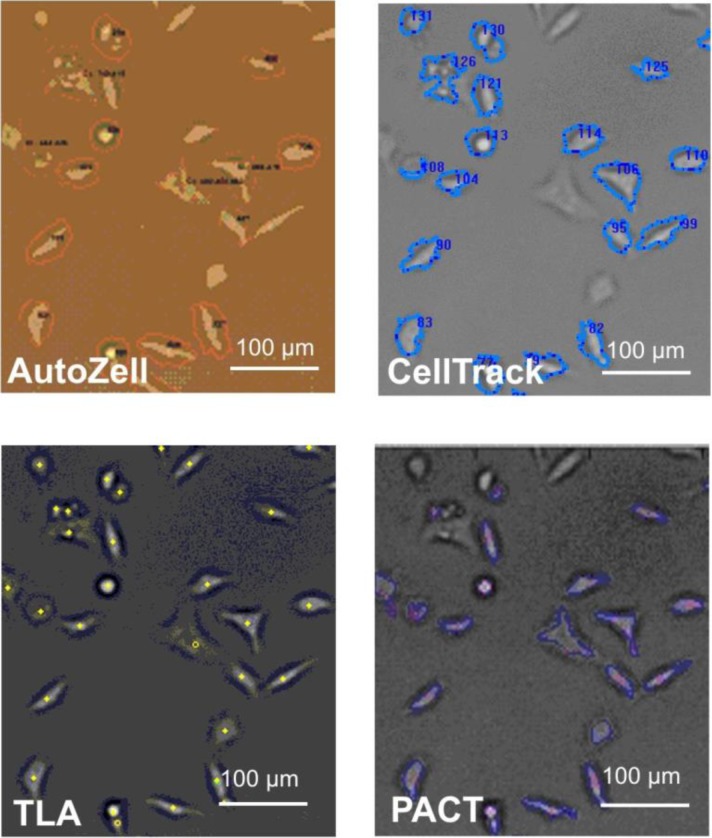
**Section of a typical frame of HeLa/glass after segmentation in Autozell, CellTrack, TLA and PACT**. Autozell shows red contours around the tracked objects, CellTrack shows blue contours around the tracked objects, TLA marks the centroid of the object with a yellow marker, and PACT shows blue contours around the tracked object and a red cross at the brightest spot in the image. The settings for each program were optimized so as to reach a balance between minimizing the number of cells missed by the tracking process and the number of false positives (segmented objects that were not cells).

The tracking efficiency was assessed by counting the objects picked in the representative images used in Table 2. A section of such a frame processed in the different programs is shown in Figure 1 for visual comparison.

### (ii) Focus Influence on Centroid Positioning

PACT was able to identify cell boundaries even when the cells were imaged slightly out of focus. Imaging out of focus in bright field microscopy has the advantage of enhancing the contrast between cells and background. This is due to light interference effects and blurring of details of cell organelles, which are visible in focus and can interfere with cell identification. We did not employ a blurring method (such as a Gaussian blur) to remove the fine-structure of cells, as that procedure would also negatively affect the sharpness of contrast between cell and background.

Long-term time-lapse recordings may lead to drift and variable focus. Another source of noise can be the light conditions and also variations in threshold and filter settings for the image processing which in PACT also can influence the cell centroid measurement. The following experiment estimates the combined effect of these influences by varying focus, which changes the cell contour and light levels.

**Figure 2:**
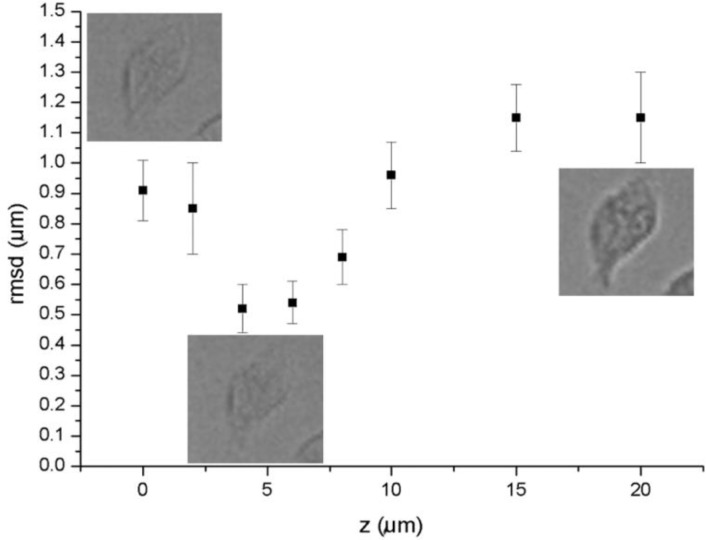
**NIH3T3 fibroblasts fixed on glass**. Plot of the root mean square displacement of the centroids from their mean in the stack of images of NIH 3T3 imaged increasingly out of focus shows the effect of defocusing on the centroid tracking accuracy. For illustration, a representative cell as viewed in the light microscope is shown at z=0 (in focus), z=4 m and z=20 m.

We imaged NIH 3T3 fibroblasts fixed on a glass slide (see GradinaruSuppInfo.zip file) in overfocus at 2, 4, 6, 8, 10, 15, and 20 µm, respectively, as well as in focus (z=0 µm), which we consider a fairly wide range of focus fluctuations for a time lapse experiment. After segmentation and post-processing, 92 cells were found in all frames. We calculated the individual cell centroids throughout the stack. From these positions we subtracted average centroid value of each frame to remove stage drift, and then evaluated the average root- mean-square displacements of all 92 cells segmented, as a function of z (Figure 2). Typical samples of segmented cells are shown in Figure 2 next to the corresponding points: z=0, 4, and 20 µm, respectively. The overall average root mean square displacement from the z-stack mean was 1.0 ± 0.1 µm.

### (iii) Precison of Centroid Positioning

In order to test the precision of the method, we compared the performance of our program with bright field images to images of dyed cells visualized by fluorescence microscopy. We have recorded images of fluorescently dyed NIH 3T3 fibroblasts fixed on glass and flat Si in both the bright field and fluorescence mode, Figure 4. 22 cells on flat Si were identified in both bright-field and fluorescence images after post-processing (see SuppInfo.zip file). A pair-wise comparison of centroids between the two imaging modes was done by translational alignment (applying a constant offset to all centroids from one imaging mode such that the mean of all centroids common to both imagining modes is exactly the same). This procedure was necessary to account for the slight light path offset due to inserting the fluorescence filter, and was followed by calculating the pair-wise RMSD of all centroids common to both imaging modes. This yielded an RMSD of 2.8 µm for the flat Si substrate (Table 3). We tested whether the orientations of the segments that connect the centroid pairs are random by plotting a histogram of the distribution of their polar angles (data not shown) which was indeed uniform within error bars. We thus conclude that the source of this discrepancy is random (white) noise.

**Table 3:** Fixed cell results. The columns *PACT* and *Manual Count* show the fraction of objects tracked after/before post-processing in bright-field images. *Co-identified* shows pairs of cells that were co-identified in fluorescence and bright field post-processed data and their corresponding RMSD

	*Brightfield cells tracked After/Before post processing*		Co-identified in fluorescence	
	PACT	Manual	Pairs	RMSD
NIH 3T3/flat Si	26 / 32=81%	35 / 42 =83%	22	2.8 µm
NIH 3T3/Si black	21 / 40=52%	40 / 48 =83%	14	4.3 µm

### (iv) Nanostructure Background Removal

To process images with grainy backgrounds, such as images recorded on a substrate of silicon black, the band-pass filter used by PACT (see materials and methods) to process images is of critical importance. By setting the high-frequency limit to 5-10 pixels, i.e., higher than the recommended value of 1-3 pixels that is appropriate for removal of pixel noise, grainy images can be analyzed. On this substrate, cells appear as shadows on a very noisy background and we were unable to detect cells with other cell tracking programs (Table 1), with the exception of TLA, which can employ a Wiener filter as a pre-processing step to cell detection.

**Figure 3:**
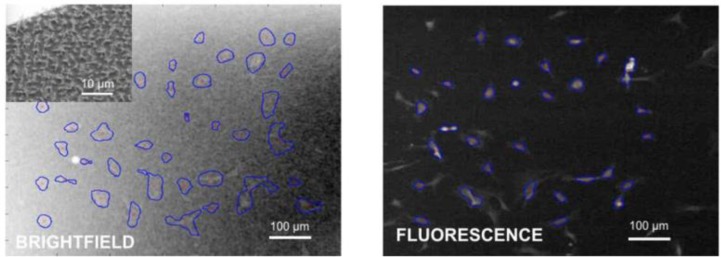
**Fixed NIH 3T3 cells on silicon black**. Left: Bright field image of NIH3T3/Si black after tracking in PACT and SEM image of the Si black substrate used viewed at a 30° angle (inset). Right: Fluorescence image of the same sample imaged in the exact same position, showing the actin cytoskeleton, also processed in PACT.

To assess the deviation in cell count and position between fluorescence and brightfield imaging, we compared the fluorescence image with the bright-field images of individual NIH 3T3 fibroblasts fixed on silicon black; see Figure 3 and Table 3. We found 14 cells co-localized after post-processing in both the bright-field and fluorescence out of 21 cells in bright-field and 25 cells in fluorescence image. The mismatch of centroid pairs was randomly oriented with uniform distribution of directions within errors due to finite statistics (data not shown). The mismatch of 4.3 µm RMSD for silicon black was larger than what was found on flat substrates 2.8 µm RMSD, as expected on these highly grainy images.

### (v) Effect of RMSD filtering

In order to remove all fixed objects automatically, an additional, RMSD-based filter is applied to the tracks prior to their analysis. For each track, the program calculates the root-mean-squared deviation of the tracked point from its mean (i.e., time averaged) value:

$$\mathrm{RMSD}=\sqrt{\frac{\sum_{i=1..N}{\left(\overset{\rho }{r_{i}}-\overset{\rho }{r_{\mathrm{mean}}}\right)}^{2}}{N-1}}$$

This is a small quantity if a cell does not move. In our experience, an RMSD threshold of approximately half a typical cell diameter (15 pixels in our images) is optimal for the exclusion of non-moving cells. This ensures that only cells that move at least one cell diameter over the course of a time-lapse experiment are used for further analyses. Most of the cells discarded this way (∼30% of all tracked immobilized objects) appear to be mechanically immobilized, attached permanently to the surface, or dead/lysed, and they should not be included in the motility analysis. The few cells that are not lysed or immobile, but simply happen to move very little for the duration of the observation, are also removed by this filter. This causes negligible error because their contribution to the velocity autocovariance function would be nearly zero if they were allowed to contribute to its statistics, while inclusion of a large number of non-moving cells and other objects would skew the statistics. Consequently, the RMSD-filter's elimination of these cells has minimal effect on our estimates for persistence times. Specifically, in our movies the RMSD filter only discarded two to three biologically relevant cells out of a full dataset of 70. We find that the convenience of having all fixed objects removed automatically greatly outweighs the negligible effect of their exclusion on statistics. In general, however, since the parameter φ_0_ is nearly equal to the mean squared velocity of the cells,one should clearly state the use of the RMSD filter and estimate its effect, since its elimination of the slow-moving cells has the potential of artificially inflating φ_0._

### (vi) Pair-wise Comparison of Tracks

Following the post-processing step, for the purpose of assessing the relative performance of the various programs with regards to centroid positioning error, we have pair-wise compared the tracks of cells tracked by each of the three programs (forming 3 datasets), TLA, Autozell, and PACT, so as to avoid cell selection bias due to the differing tracking methods implemented. The difference between the tracks of the same cell obtained with two different tracking programs is due to a conglomerate of errors arising from of differences in the spatial filtering, thresholding and tracking algorithm employed by each program. To remove systematic error, the tracks common to all three datasets (the subsets) were first translationally aligned to minimize the pair-wise RMSD (Eq. 3) of the three subsets. This minimal pair-wise RMDS was used as a measure of the mismatch between the coordinates output by each program.

$$\text{RMS}{\text{D}}_{\text{j},\text{k}}=\sqrt{\frac{\sum_{\text{i}=1..\text{N}}{\left({\text{X}}_{\text{i}}^{\text{PROGRAM}\_\text{j}}-{\text{X}}_{\text{i}}^{\text{PROGRAM}\_\text{k}}\right)}^{2}}{\text{N}}}$$

The magnitude of pair-wise mismatches between the three programs may be found in Table 4 (off-diagonal elements). We attribute the higher RMSD between the Autozell subset and the subsets generated by the other two programs to the fact that Autozell rounds the coordinates of centroids to the nearest integer, which necessarily introduces an additional random error. Also note that the default settings of TLA, which have been used in this and the following section unless explicitly stated otherwise, involve a special smoothing procedure of the tracks which slightly alters centroid coordinates (see section vii below).

**Table 4:** Positional error effects. The diagonal elements show the centroid positional measurement error as determined from a fit to Fürth's formula. The off-diagonal elements show the pair-wise RMSD between the programs. All values in µm.

Positional error/µm	PACT	TLA	Autozell
**PACT**	1.40 ± 0.05	1.9	2.6
**TLA**	-	0.36 ± 0.03	2.7
**Autozell**	-	-	1.36 ± 0.04

### (vii) Centroid Positional Error

We have also evaluated the mean square displacements of cell centroids as a function of time for each of the three subsets to assess the centroid positional error for each of the three cell tracking programs. By fitting to the extended Fürth's formula (Supporting information Sect. 7), we determined the centroid positional measurement error, σ_pos_. These values are listed in Table 4.

The results in Table 4 are interpretable in light of Equation 3 which relates each tracking algorithm's intrinsic noise (denoted as the white noise term ξ_i_ with variance 2σ_pos,i_^2^) with the measured centroid coordinate value r_i_. The i subscript refers to the algorithm/program used:

$${\vec{r}}_{i}={\vec{r}}_{i}^{\mathrm{true}}+{\vec{\xi }}_{i}$$

In the limit of large N, the measured value of the centroid is biased by the choice of algorithm due to positional noise (random error, variance 2σ^2^_pos,i_), as well as any systematic, algorithm-specific, centroid positioning error. Thus the pair-wise RMSD is:

$$\text{RMS}{\text{D}}_{\text{i},\text{j}}^{2}=\langle {\left({\vec{r}}_{i}-{\vec{r}}_{j}\right)}^{2}\rangle =\langle {\left({\vec{r}}_{i}^{\text{true}}-{\vec{r}}_{j}^{\text{true}}\right)}^{2}\rangle +2\sigma_{\text{pos},i}^{2}+2\sigma_{\text{pos},j}^{2}+\left(\text{cross terms}\right)$$

Indeed, the difference (-0.6 to 3.3 µm^2^) between the mean squared pair-wise displacement (square of RMSD from Table 4) and the corresponding sum of the variances () is very similar to the variances themselves. Thus, the relative centroid tracking accuracy of the different programs does not differ significantly from the positional noise level itself.

While Autozell and PACT appear to track cells with similar positional noise levels, the TLA dataset has a much lower positional noise. We attribute this effect to the Kalman filter employed for noise reduction and subsequent smoothing of cell tracks using a moving average filter as provided in the standard procedure for processing bright field images in TLA[33]. Indeed, reprocessing this movie in TLA with the moving average filter switched off and the Kalman filter adjusted so that no prediction is made, yields σ_pos_=0.61 ± 0.07 µm, nearly twice that determined with this program's standard procedure, but still lower than PACT and Autozell's.

### (viii) Reproducibility of Statistical Data Analysis

**Figure 4:**
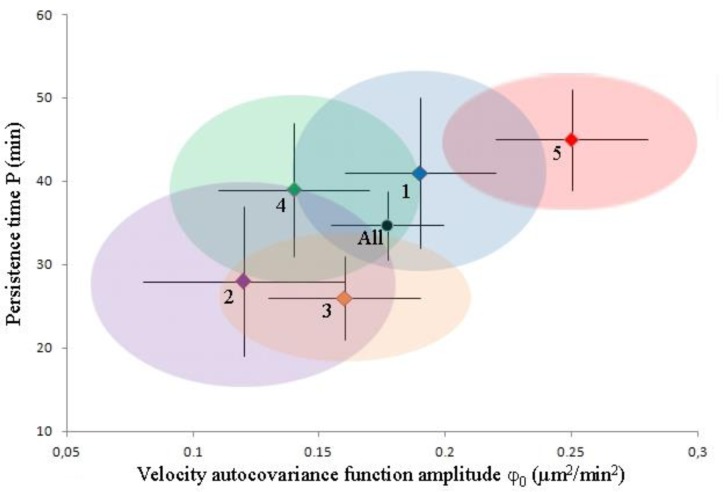
**Motility parameters for NIH 3T3 cells on glass analyzed with PACT**. Each of the 5 movies is depicted as a point in the associated φ_0-_P space. The black error bars show the overdispersion-*un*corrected 1σ (i.e. original fit results), while the corrected 1σ levels are shown as color-matched ellipsoids around each point. Color code: blue - movie 1 (9 tracks after post-processing); purple - movie 2 (12 tracks); orange - movie 3 (15 tracks); green - movie 4 (8 tracks); and red - movie 5 (14 tracks). The black point shows the weighted mean of parameters from the 5 movies and corresponding overdispersion-corrected 1σ error bars.

Before testing whether different cell tracking programs yield the same velocity autocovariance statistics for the tracks that they find for a given movie of motile cells, we assessed the reproducibility of the results obtained using PACT. Five movies were recorded on five different positions on the same sample and each movie and the combined dataset were statistically analyzed. PACT found 8 to 15 tracks per movie, each movie (Supporting info. Sect 4) recording ∼12 hours of NIH 3T3 motility on glass, with ▵t=2 min between successive frames. Results are shown in Figure 4 below and in Table S1.

The 5 pairs of (P, φ_0_) parameters were determined from a weighted least-squares fit to velocity autocovariance function data points. We note that the error bars of the autocovariance data points, shown in Supp. info. Figure S2, become increasingly underestimated as recording time goes by, due to data redundancy in the method used to calculate them (Supp. info. Eq. S1). This leads to artificially low weighing factors in the least-squares algorithm, which lowers the error bars of the fit parameters P and especially φ_0_. Additionally, the autocovariance data points themselves are correlated (i.e. not statistically independent) as they are calculated from the same set of cell centroid coordinates. This artificially deflates the error bars of the fit parameters P and φ_0_ (fit results reported in Figure 4 and Supp. info. Table S1). To account for these data overdispersion effects while assessing the reproducibility of our results, we quantitated the degree of this artifact in our data (see Supp. info. Sect. 6). This procedure allows the estimation of the motility parameters from all 5 movies as a weighted average of the parameters measured from individual movies:=35 ± 4 min and=0.18 ± 0.02 µm^2^/min^2^. This matches nearly exactly the parameters determined by combining the 5 sets of centroid coordinates and analyzing those coordinates as one set (“combined dataset,” Table S1, Supporting information): P=36 ± 4 min and φ_0_=0.18 ± 0.02 µm^2^/min^2^. We conclude that the observed discrepancy between the motility parameters determined from the five movies is statistically insignificant: 4/5 parameter pairs match within 1σ (68.3% expected to match) after correction. This shows that this method of analysis is reproducible.

### (ix) Comparing Velocity Autocovariance Functions

**Figure 5:**
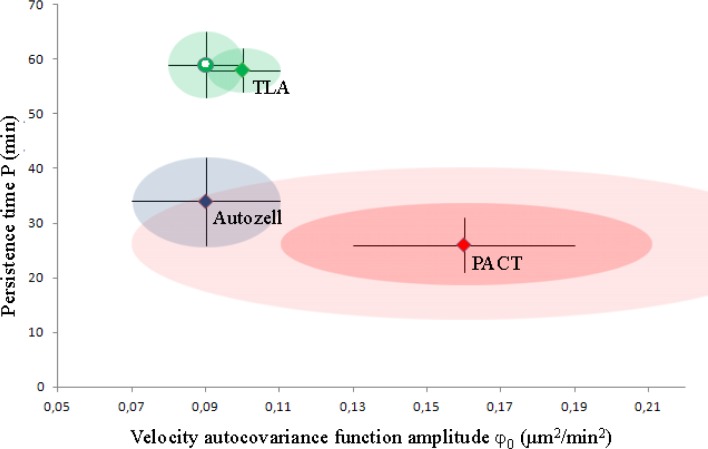
**Comparison of results obtained, respectively, with PACT, Autozell, and TLA for two parameters of a typical analysis of cell motility**. Each of the datasets is depicted as a point in the associated φ_0-_P space. The black error bars show the overdispersion-*un*corrected 1σ (i.e. original fit results). Both the corrected 1σ (red ellipsoid) and 2σ levels (light red ellipsoid) are shown around the PACT point. In the absence of overdispersion information, the *un*corrected 1σ levels are shown as color-matched ellipsoids around the Autozell (blue) and TLA (green) points. The Kalman-filtered and moving-average smoothed P-φ_0_ point is shown as a green hollow circle, to distinguish it from the unfiltered, unsmoothed result, shown as a green square.

We have compared the accuracy of various cell tracking programs by testing them on NIH 3T3 cells recorded on a glass substrate (movie in Gradinaru.zip). The cells in this movie were tracked by Autozell, MATLAB, and TLA, and following post-processing, the tracks found by each of the three programs (the original datasets) were analyzed. We have named the resulting tracks the *original datasets*: fifteen tracks identified by PACT following post-processing, nine tracks identified by Autozell, and fourteen tracks identified by TLA using the default settings. We have also included the motility parameters obtained from TLA with the moving average smoothing option turned off and adjusted to prevent any Kalman filter prediction (“TLA/raw”) such that the position measurement is accepted as the true object position (fifteen tracks). The results are shown in Figure 5 and in Supp. info. Sec. 5.

The motility parameters thus determined with Autozell and/or TLA differ substantially (φ_0_ nearly halved, P nearly doubled) from the results obtained using PACT. The Autozell and PACT results are statistically equivalent as there is a clear overlap between the blue (Autozell, uncorrected 1σ) and light red ellipsoid (PACT, corrected 2σ), and a possible overlap within 1σ between the two programs if the Autozell results were data overdispersion-corrected. However, both TLA ellipsoids (1σ) remain well outside the range of overlap with the other two programs. We speculated that the Kalman filter or the moving average filter was the culprit; however, the results do not change much upon removal of these effects. This is perhaps the best illustration of the increase in fit imprecision due to the correlation of the velocity autocovariance data points. Indeed, the velocity autocovariance functions determined using the TLA datasets show significant structure (Supp. info. Fig S3,), which would indeed lead to significant underestimation of the error bars, manifested as the substantially smaller green ellipsoids in Figure 5. We are uncertain of the cause of the high correlation of the velocity autocovariance values as determined with TLA.

**Figure 6:**
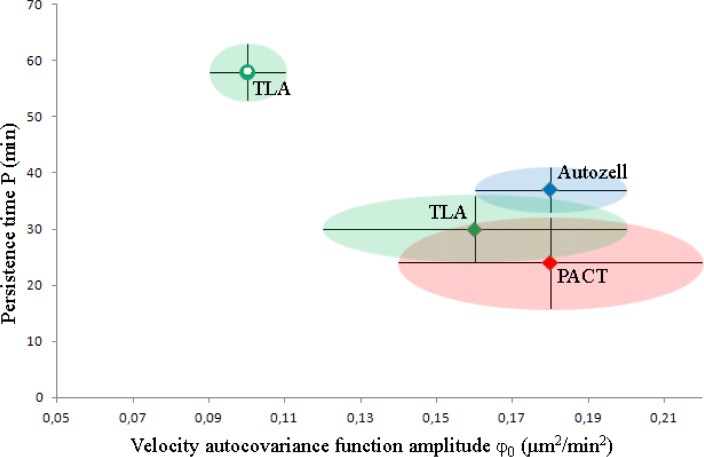
**Comparison of PACT, Autozell, and TLA for the purpose of assessing cell motility analysis on identical tracks**. Each of the subsets is depicted as a point in the associated φ_0-_P space. The black error bars show the overdispersion-*un*corrected 1σ (i.e. original fit results). In the absence of overdispersion information, the *un*corrected 1σ levels are shown as color-matched ellipsoids around the PACT (red), Autozell (blue), and TLA (green) points. The Kalman-filtered and moving-average smoothed P-φ_0_ point is shown as a green hollow circle, to distinguish it from the unfiltered, unsmoothed result, shown as a green square.

To account for the possible effects of not having used the tracks of the exact same cells in this analysis, we have also separately analyzed the common subset of tracks of the cells detected by all three programs (hereafter referred to as the subset). The results are shown in Figure 6 below and in Supp. info. Sec. 5.

Only three of the tracks correspond to cells that were identified and tracked by all three programs. A comparison of cell motility parameters determined from raw data (i.e. without any filtering/smoothing procedure applied to the data) in Figure 6 shows that there is no significant variation in the motility parameters, φ_0_ and P. We conclude that the observed discrepancy between the motility parameters determined with the three programs is statistically insignificant: 2/3 persistence times match within 1σ and all (3/3) φ_0_ parameters do as well (at most 68% expected to match), despite the shown error bars being underestimates of the real error bar levels.

We note, however, that the Kalman filtered- and moving average smoothed-subset yields a very different P-φ_0_ set of results. This is at least in part due to the substantial underestimation of the respective error bars, owing to the highly structured velocity autocovariance functions (Supp. Info. Fig. S3). Finally, to test the the possible effect of Autozell's tracking method, which involved truncating the centroid coordinate to the nearest pixel, we have similarly rounded the coordinates generated by PACT to the nearest pixel to generate the PACT/cut common dataset (Supp. Info. Table S2). We found that the motility parameters determined from this subset had not changed, and conclude that the systematic centroid truncation performed by Autozell is unlikely to affect the motility analysis results.

These observations raise the point of the importance of the definition of the cell centroid and the effect that this definition has on motility data. We elaborate on this below.

## Discussion and conclusion

The segmentation efficiency tested in (i) was performed by four programs listed in Table 2. These programs perform reasonably well on flat substrates with some detection efficiencies being above 90% of the manual count. These values were obtained by one user optimizing settings and may vary between uses and time lapse movies. Image filtering routines were shown to be essential for efficient detection of cells on structured backgrounds (iv), and these routines are not available with all programs (Table 1). Additionally, settings may need to be optimized over time in a movie (ii) due to variations in imaging conditions. This may be aided by the linear interpolation possible in PACT.

Different programs select somewhat different cells and hence do their respective measurements and statistics for somewhat different overlapping subpopulations. This amounts to filtering the original cell population with somewhat different filters, with the preferences of the segmentation algorithm of each program providing a different filter. The variations in segmentation combined with track formation algorithms such as the RMSD filter (v) can result in very different subpopulations of tracks from the same data set. This is borne out by the fact that only three trajectories were selected by all tracking programs. A single program may be prone to biased selection of subpopulations of cells with specific features, such as proximity to other cells and specific morphology features, all depending on the algorithms being used, sample details and users choice of settings. For these reasons, we recommend that before performing automated time-lapse analysis on a specific movie, the user should assess how well the program performs compared to manual counting and consider carefully if the undetected cells might result in a bias in the cell subpopulation tracked by the software.

We have developed PACT for tracking cells on non-uniform backgrounds. To that end, we tested PACT on silicon black substrates. Cells on such substrates are often difficult to distinguish from the background even by eye. TLA and PACT are the only programs that successfully tracked cells in these images. The Wiener filter that TLA employs in its bright field tracking method, is comparable to the convolution filter implemented in PACT with regard to cell recognition. However, we have specifically designed PACT to reduce operator effort at the post-processing step, which is the most time-consuming step of the analysis. We accomplished this by implementing additional automated post-processing features: restrictions on the area of the object and minimal inter-cell distance. These features make PACT less likely to find false positives and this is reflected in the percentage of objects that pass the post-processing step of PACT in comparison with TLA: 81% versus 72% for flat Si and 52% versus 31% for silicon black. We conclude that PACT is more useful for the sparse cell culture motility analyses that we perform in this paper. PACT can, however, be less efficient for other applications such as dense cell cultures.

The positional error was shown in Section (vii) to be of the order 1-2µm. Compared to this noise level, the cell centroids appear to be fairly reliably determined: They are not strongly influenced by variations in focus and threshold settings, but scatter with an RMSD of 1µm for a 20 µm focus variation in the test performed in Section (ii). The brightfield image centroid position correlates with the fluorescence microscopy results with an RMSD on flat surfaces of 2.8µm as shown in (iii), while nanostructured substrates increase the RMSD to 4.3µm. This indicates that bright field microscopy can be used for motility analysis with precision comparable to that of fluorescence microscopy and that the overall process is not strongly influenced by variations in the experimental procedure and image segmentation.

After removal of positional noise effects, the pair-wise RMSD of the tracks compared in Section (vi) are similar to the positional noise level itself. That is also supported by our experiments introduced in sections (iii and iv), which show that the PACT centroids measured from bright-field images are comparable to a “golden standard” of fluorescence images of the same samples. These results indicate that the different programs can be used to assess cell centroids reliably (accurately and precisely) from bright-field images.

We tested the internal consistency of the velocity auto-covariance function measurements on cell motility in Section (viii) and found that the results are consistent, especially when accounting the artificial underestimation of the motility parameters’ error bars due to data overdispersion. Comparing the motility parameters obtained with different programs in (ix), Figure 5 and 6, we find that the different programs give statistically consistent results when used on the same tracks in Figure 5. PACT and Autozell provide motility parameters that agree well within their respective uncertainty, when data overdispersion effects are accounted for. However, TLA displays unusually high correlations in the velocity autocovariance function values and subsequent under- or over-estimation of motility parameters.

The motility parameters obtained from different programs in Fig 5 scatter to not achieve full mutual agreement within error bars. This is even with due consideration of the data overdispersion effects in Section (viii) that provide larger error bars. Given the agreement in Fig 6, this deviation must originate in the way the programs vary in segmentation. This result indicates that one must always include careful documentation of the way that a program segments an image into cells that will be used for motility analysis.

Finally, we have implemented the features present in PACT (RMSD filter, track screening) in TLA.

In conclusion, our study shows that cell tracking can be done with different programs, often with reasonable segmentation efficiency and precision: the pair-wise RMSD of tracks output by two different programs is 2-3µm, consistent with a positional noise per dataset of ∼1µm. Our analysis of results for the velocity auto-covariance function demonstrates that different programs provide comparable precision in the motility parameters P and φ_0_ in a statistically significant manner when all sources of errors are accounted for, but comparison between data obtained with different programs should be done with caution, since different programs well may select different cell subpopulations based on their segmentation algorithm. For nanostructured substrates this is can be important, as they appear to make cells display a wide phenotypic variability [35]. Comparison of data obtained with different programs doing automated analysis is hence difficult, unless detailed tests have been done for how each program performs in segmentation and how its results compare to those of other programs.

## Supplementary Material

Supporting Information Document for “Assessment of Automated Analyses of Cell Migration on Flat and Nanostructured Surfaces”

Supplementary Movies
