# Supplementary material for: Assessment of Automated Analyses of Cell Migration on Flat and Nanostructured Surfaces
Source: Comput Struct Biotechnol J. 2012 Nov 21;1:e201207004. doi: 10.5936/csbj.201207004 (PMC3962212; doi:10.5936/csbj.201207004)
Supplement: Supporting Information Document for “Assessment of Automated Analyses of Cell Migration on Flat and Nanostructured Surfaces” [file CSBJ-1-e201207004_SM0001.pdf]

Supporting Information Document for “Assessment of Automated Analyses of Cell Migration on Flat and Nanostructured Surfaces”

Cristian Grădinaru[1§], Joanna M. Łopacińska[1], Johannes Huth[2,3], Hans A. Kestler[2,4], Henrik Flyvbjerg[1], Kristian Mølhave[1§]

Contents

1 Overview of supplementary files 1
2 Supplementary materials and methods 2
3 Fits to Experimental Data 3
4 Motility parameters for 3T3/glass 4
5 Comparison of PACT, Autozell, and TLA 6
6 Data overdispersion correction 10
7 Modified Fürth’s formula fits 10
8 Typical PACT settings 12
References 12

1 Overview of supplementary files

The SuppData.zip file contains

- track_movie_ver_1_0.m + rgb.m + bpass.m
  MATLAB .m files containing the source code of PACT
- setup_brigthfield_tracking.mat :
  TLA settings file, File that can be used to set the parameters in TLA as used in the paper.
- BF z-**.*.tif:
  Focus z-stack series with the filenames number indicating the z-level in µm.
- Si FL.jpg and Si BF.jpg
  Fluorescnece and Bright field images of 3T3 cells on flat Si substrate.
- Si black FL.jpg and Si black BF.jpg
  Fluorescence and Bright field images of 3T3 cells on Si black substrate
- HeLa-Glass.tif
  Brightfield image of HeLa on Glass substrate

Movie files.

- SuppMovie1-5.zip
  Contains movie *.avi: 5 movies of 3T3 cells on glass substrate. Δt=2 min between successive frames.

2 Supplementary materials and methods

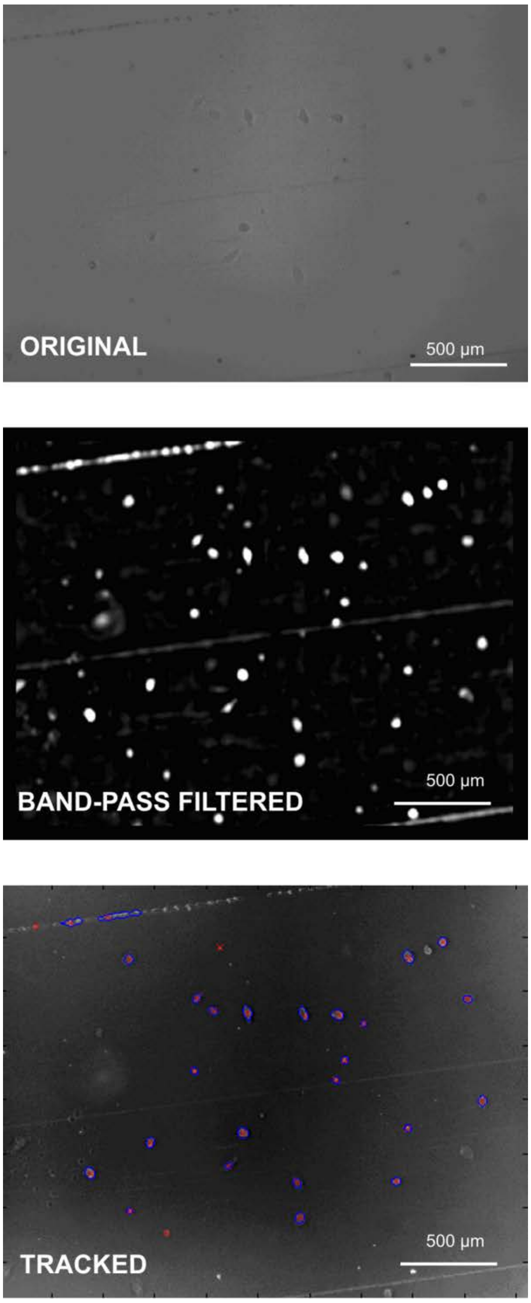

Figure S1 - Image segmentation in PACT: band-pass filtering followed by segmentation by thresholding and edge detection. Image of HeLa/glass (top, available in SuppData.zip), band-pass filtered using frequency limits of 3 and 30 pixels (middle), and processed frame (bottom) showing peaks brighter than peak-exclusion threshold of 60 as red crosses and cell contours as blue shapes. Some peaks do not have a blue contour around them, as they did not pass the additional cell selection criteria (size and proximity to other cells).

3 Fits to Experimental Data

We provide below the defining equation of the velocity autocovariance function (<…> denotes ensemble averaging):

$$\varphi(t) = \left\langle \vec{v}(t) \cdot \vec{v}(0) \right\rangle$$

and the formula that has been used to calculate the experimental velocity autocovariance function from the motility data:

$$\varphi_j^{exp} = \frac{\sum_{\alpha=1}^{\text{last track}} \sum_{k=0}^{N_\alpha - j} \vec{u}(t_j + t_k) \cdot \vec{u}(t_k)}{\sum_{\alpha=1}^{\text{last track}} N_\alpha} \qquad \text{Equation S1}$$

Here N_α denotes the number of points in track α, $t_j = j\Delta t$ and $t_k = k\Delta t$ as all of our movies were recorded at a fixed frame rate, and u(t) are secant (frame-to-frame) velocities, defined as the displacement of the cell centroid from one frame to the next divided by the time interval that separates the two frames (Δt). This is different from the instantaneous velocity v(t) due to discretization and positional noise.

4 Motility parameters for 3T3/glass

Figure S2 -
Left: Monoexponential fits (red) to the velocity autocovariance function of NIH 3T3 cells from movies in SuppMovie1-5.zip, shown as black squares in the graphs below (fit results shown in figure 4 and Table S1). Right: Red trace showing fits to 10-point bins of the velocity autocovariance function data points, shown in black.
The two columns present raw and binned data. The right column with binned data shows that the fit, which represents the expected value of the raw data as function of lag time, actually agrees with the mean value of the raw data, again as function of lag time. This is not clearly seen from the raw data sets alone, and the agreement is here demonstrated for each independent set of raw data.. The rows present the analysis of five independent datasets acquired under identical conditions to provide 5 independent measurements of the autocovariance function for this population. These independent measurements are used to obtain five independent fits of the monoexponential autocovariance function. From the resulting five independent fitted parameter sets we can calculate the mean values of P and f_0 and thus estimate true standard errors on these values.
The motility parameters measured by fit to the binned autocovariance function match extremely well the ones shown in Table S1.

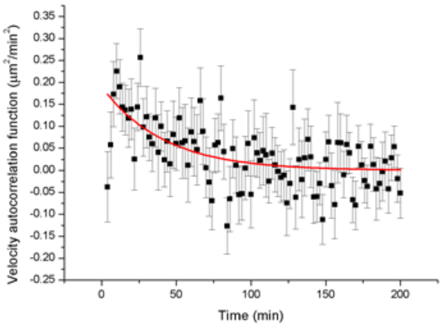

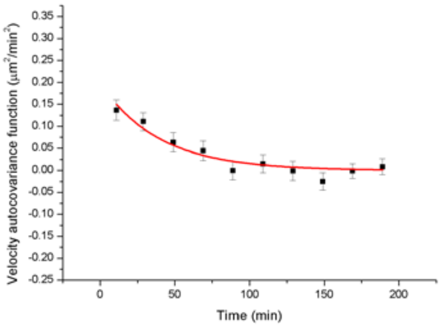

Movie 1

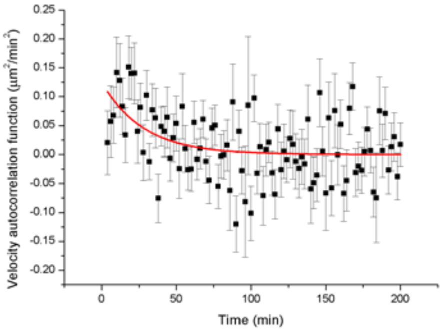

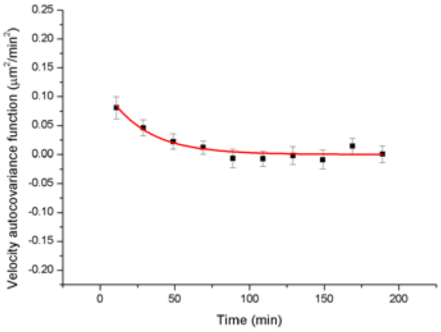

Movie 2

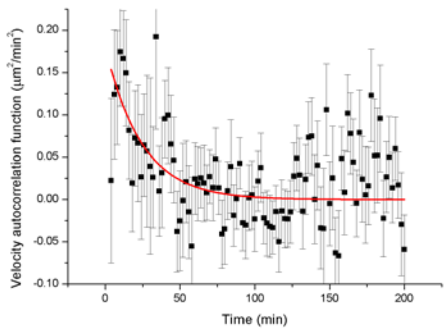 Movie 3	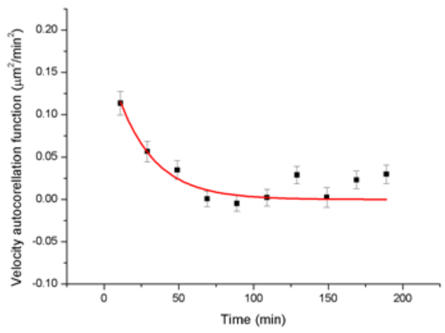
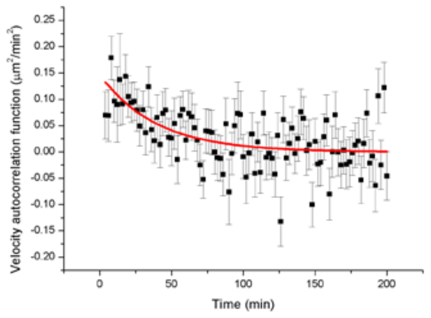 Movie 4	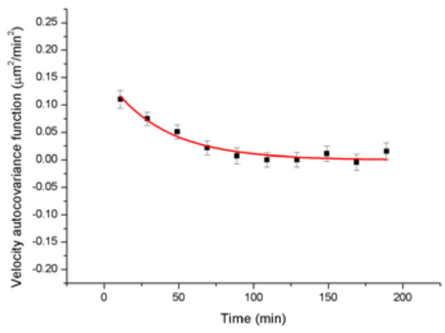
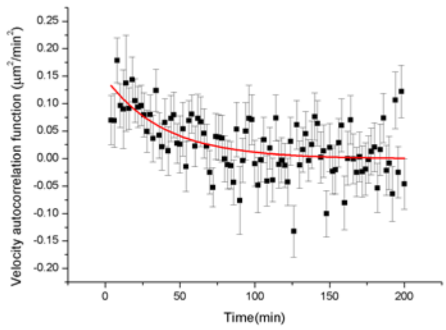 Movie 5	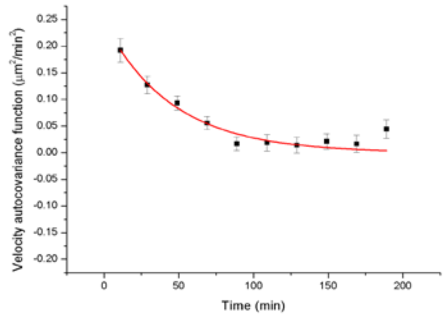
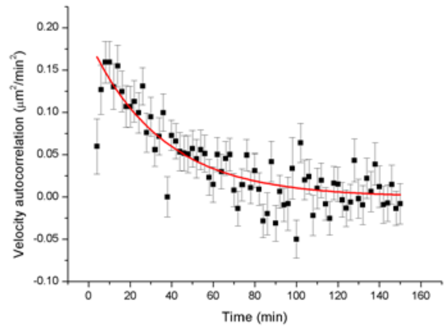 Combined dataset	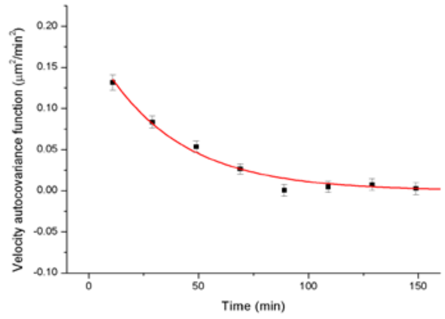

Table S1 - Motility parameters for 3T3/glass (9 tracks remaining after post-processing from movie

1, 12 tracks from movie 2, 15 from movie 3, 8 from movie 4, and 14 from movie 5; the combined datasets includes the data from all these movies: 58 tracks).

Movie #	φ_0 / baseline: $\mu m^2/min^2$	$\sigma_{\varphi 0}^{corr}$: $\mu m^2/min^2$	P: min	σ_P^{corr}: min
Combined	0.18 ± 0.02 / 0.003 ± 0.004	-	36 ± 4	-
1	0.19 ± 0.03 / -0.02 ± 0.02	0.05	41 ± 9	12
2	0.12 ± 0.04 / -0.002 ± 0.008	0.06	28 ± 9	12
3	0.16 ± 0.03 / 0.009 ± 0.004	0.05	26 ± 5	7
4	0.14 ± 0.03 / -0.001 ± 0.009	0.05	39 ± 8	11
5	0.25 ± 0.03 / 0.014 ± 0.009	0.05	45 ± 6	8

5 Comparison of PACT, Autozell, and TLA

Figure S3 - Left: Monoexponential fits (red) to the velocity autocovariance function of NIH 3T3 cells from movie #1 in SuppMovie1-5.zip, shown as black squares in the graphs below (fit results shown in figure 5 and Table S2). Right: Right: Red trace showing fits to 10-point bins of the velocity autocovariance function data points, shown in black.

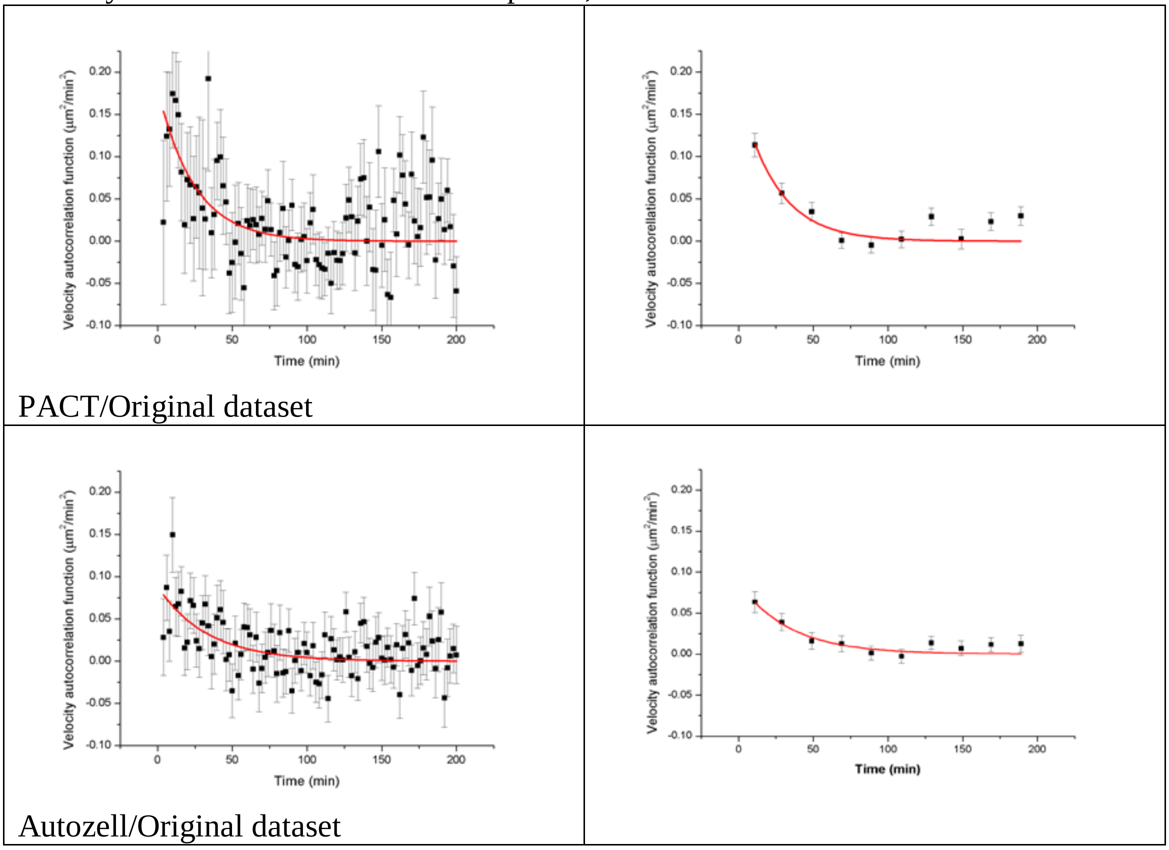

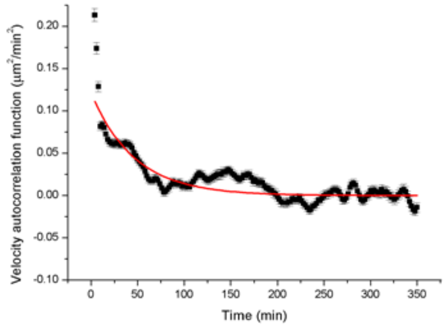

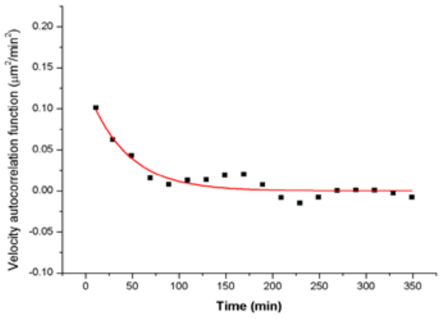

TLA/Original dataset

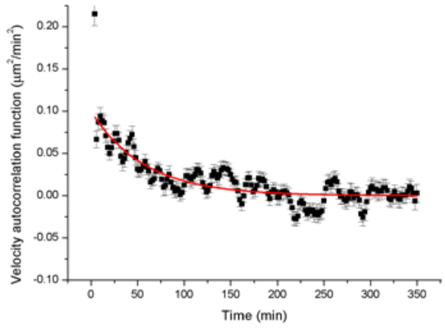

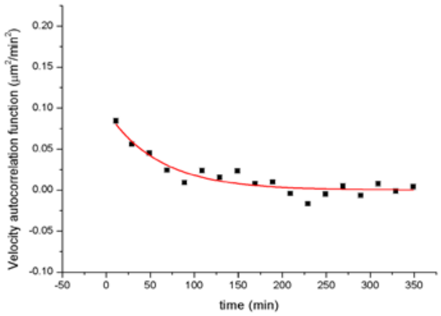

TLA:no filter-smoothing/Original dataset

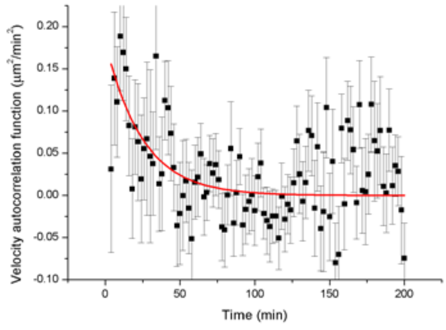

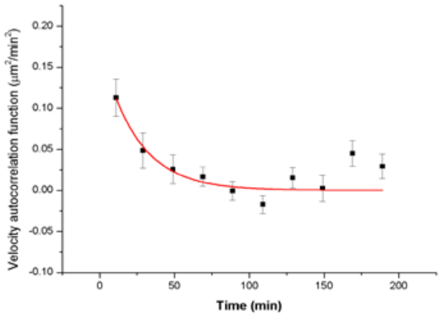

PACT/subset

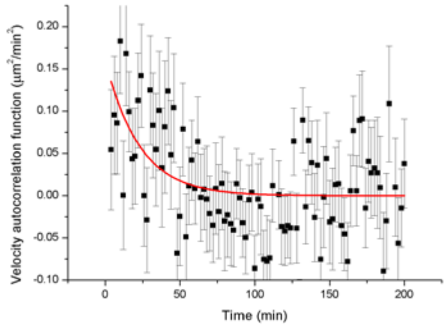

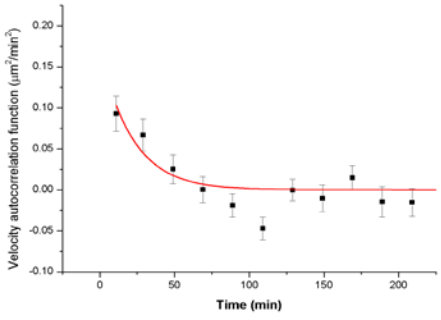

Autozell/subset

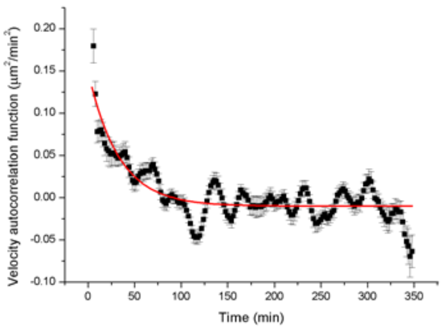

TLA/subset

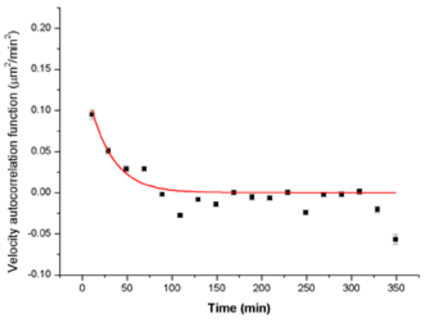

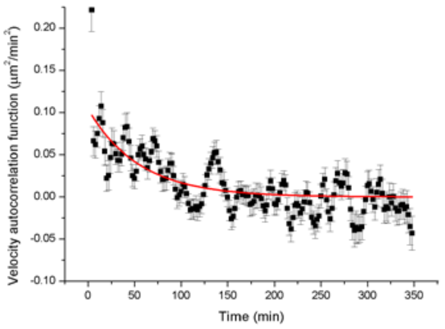

TLA/subset/no filter-smoothing

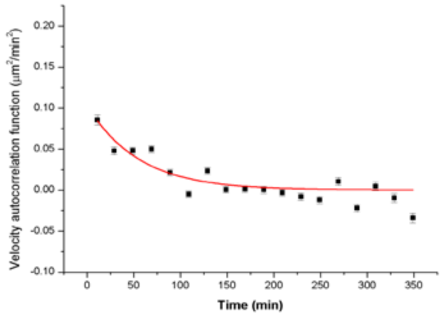

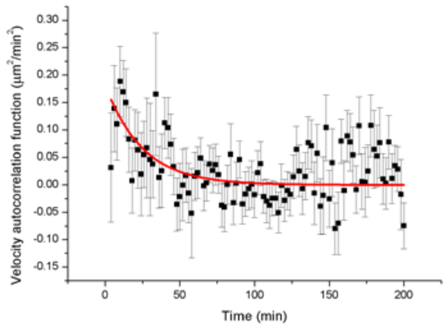

PACT/subset/nearest digit-truncated

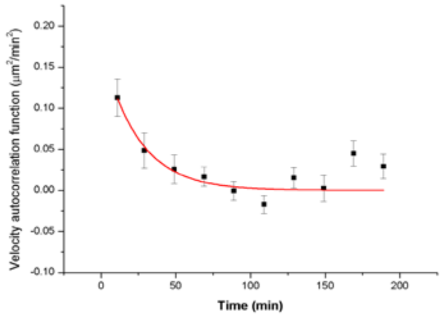

Table S2 - Comparison of PACT, Autozell, and TLA with regards to data analysis

Program	φ_0 ($\mu m^2/min^2$)	P (min)
PACT	0.16 ± 0.03	26 ± 5
Autozell	0.09 ± 0.02	34 ± 8
TLA/Kalman filter/MA smoothing	0.09 ± 0.01	59 ± 6
TLA/no filter/no smoothing	0.10 ± 0.01	58 ± 4
PACT subset	0.18 ± 0.04	24 ± 6
Autozell subset	0.18 ± 0.02	37 ± 4
TLA subset/no filter/no MA	0.16 ± 0.04	30 ± 4
TLA subset/filter/MA	0.10 ± 0.01	58 ± 7
PACT subset/truncated	0.18 ± 0.04	25 ± 6

6 Data overdispersion correction

We have initially determined the weighted mean and standard deviation of the mean for the motility parameters P and φ_0 from the 5 NIH 3T3/glass movies (Table S1, Figure S1, Supporting information 1) using the standard formulas (N is the number of movies, in this case 5; equation shown for P and similar for φ_0):

$$\bar{P} = \sum_{i=1}^{N} \frac{P_i}{\sigma_i^2} \Big/ \sum_{i=1}^{N} \frac{1}{\sigma_i^2} \quad \text{and} \quad \sigma_{\bar{P}}^2 = 1 \Big/ \sum_{i=1}^{N} \frac{1}{\sigma_i^2}$$ Equation S2

A correction is due to account for data overdispersion in the individual P_i values. This method assumes that the data is drawn from distributions of the same mean P value (which is sensible given the data in figure 4 is from the same sample), but with different σ_i values (also a reasonable assumption given the differing number of tracks and track lengths included in each of the 5 movies in figure 4). The χ^2 (quantity being minimized as part of the least-squares fit) is expected to be 1 for data dispersed in accordance to the provided σ_i's, but that is not the case for this dataset: χ^2 (P) = 1.8 and χ^2 (φ_0) = 2.5. Thus, we correct the error bar of the mean P value above:

$$\sigma_{\bar{P}}^{corr} = \sqrt{\chi^2(P)} \cdot \sigma_{\bar{P}}, \quad \text{with} \quad \chi^2(P) = \frac{1}{N-1} \sum_{i=1}^{N} \frac{\left(P_i - \bar{P}\right)^2}{\sigma_i^2}$$ Equation S3

In the absence of additional information, we have assumed equal overdispersion for all 5 movies and thus estimated the real dispersion of the motility parameters for an individual movie:

$$\sigma_{P_i}^{corr} = \sqrt{\chi^2(P)} \cdot \sigma_{P_i}$$ Equation S4

7 Modified Fürth's formula fits

Fürth's formula [1] for the mean square displacement of a motile cell has been routinely used to describe cell motility. On a two-dimensional surface, this takes the following form:

$$\left\langle \left(\vec{r}(t) - \vec{r}(0)\right)^2 \right\rangle = 4D\left(t - P\left(1 - e^{-t/P}\right)\right)$$ Equation S5

Here $\langle \; \rangle$ denotes expectation value, $\vec{r}$ is the trajectory vector, and t is time. D and P are the motility parameters of the organism which, in the context of a standard OU process, are the diffusion coefficient and the persistence time, respectively. Fürth's formula, however, is not limited to motility models following the standard OU process[2]; instead, it is generally applicable to motility models whose velocity autocorrelation function follows a simple mono-exponential dependence of the type encountered with the fibroblasts studied in this paper.
Here we expand Fürth's formula to account for the effect of positional noise on the motility data:

$$\left\langle \left(\vec{r}_{exp}(t) - \vec{r}_{exp}(0)\right)^2 \right\rangle = \left\langle \left(\vec{r}(t) - \vec{r}(0) + \vec{\xi}_t - \vec{\xi}_0\right)^2 \right\rangle = \left\langle \left(\vec{r}(t) - \vec{r}(0)\right)^2 \right\rangle + 4\sigma_{pos}^2$$

thus:

$$\left\langle \left(\vec{r}_{exp}(t) - \vec{r}_{exp}(0)\right)^2 \right\rangle = 4D\left(t - P\left(1 - e^{-t/P}\right)\right) + 4\sigma_{pos}^2$$ Equation S6

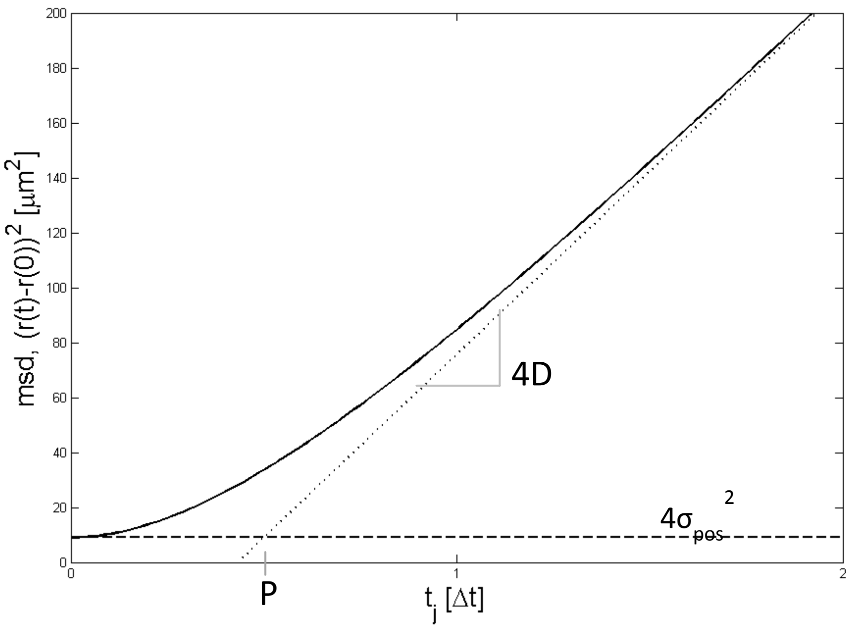

Figure S4: Fürth’s formula for the mean squared displacement of persistent random motion described by the OU-process. Full line: Mean squared displacement from equation S6; Dotted line: Asymptotic behavior at t→∞ with slope 4D. Dashed line: Asymptotic behavior at t→0. The dashed line intersects the msd axis at $4\sigma_{pos}^2$. The dotted and dashed lines intersect at t = P.

Fitting a plot of mean square displacement of a motile cell to Equation S6 provides a reliable measure of the positional noise. To test this, the trajectories of 50 independent motile cells following a standard OU process with parameters typical of fibroblasts have been simulated over 2 hours as described in Appendix D of [3], yielding experimental-like data from 128 frames (data not shown). While the estimates for P and D are imprecise (25% error for P, 28% for D), the positional error can be determined remarkably accurately from this fit (0.9978 ± 0.0008 µm vs. 1 µm input). This is because the error bars of $\left(\vec{r}_{exp}(t)-\vec{r}_{exp}(0)\right)^2$ are correlated, thus the fit to Fürth’s formula is not correctly weighed. As an asymptote of Fürth’s formula as t→0, σ_{pos} can be determined much more accurately.

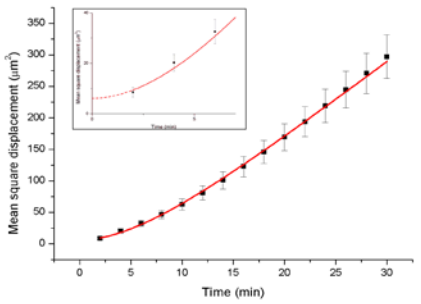

PACT

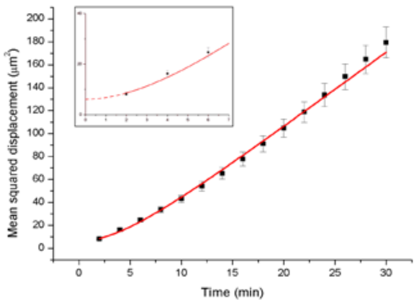

Autozell

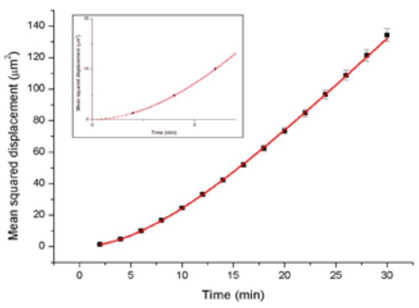

TLA

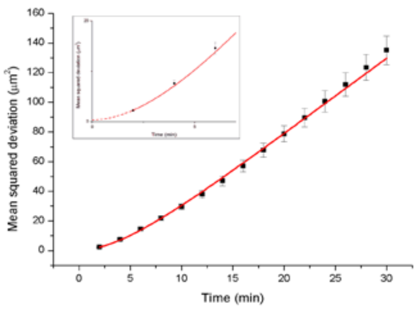

TLA/no filter

Figure S5 - Modified Fürth’s formula fits (red) to mean square displacement of NIH 3T3 cells movie 1, shown as black squares in the graphs below (fit results shown in section (viii) of this paper). The insets show the first five points of the mean squared displacements and the corresponding fit, extrapolated to zero (dotted line). The y intercept is used to determine the positional noise.

8 Typical PACT settings

These setting are the typical settings we have used in PACT, exemplified by movie 2 from the set of 5 movies..

**Parameters**	**Movie 2**	**Typical range**
Peak exclusion threshold	60 of 255	30-100
Contour cutoff	40%	40-80%
RMSD threshold	7.5 pixels	0-20

References

[1] R. Fürth, “Die Brownsche Bewegung bei Berücksichtigung einer Persistenz der Bewegungsrichtung. Mit Anwendungen auf die Bewegung lebender Infusorien,” *Zeitschrift für Physik*, vol. 2, no. 3, pp. 244-256, Jun. 1920.

[2] G. E. Uhlenbeck and L. S. Ornstein, “On the Theory of the Brownian Motion,” *Phys. Rev.*, vol. 36, no. 5, pp. 823-841, 1930.

[3] S. F. Nørrelykke and H. Flyvbjerg, “Power spectrum analysis with least-squares fitting: Amplitude bias and its elimination, with application to optical tweezers and atomic force microscope cantilevers,” *Review of Scientific Instruments*, vol. 81, no. 7, pp. 075103-075103-16, Jul. 2010.
