# Supplementary material for: Assessment of Automated Analyses of Cell Migration on Flat and Nanostructured Surfaces
Source: Comput Struct Biotechnol J. 2012 Nov 21;1:e201207004. doi: 10.5936/csbj.201207004 (PMC3962212; doi:10.5936/csbj.201207004)
Supplement: Supplementary Movies [file CSBJ-1-e201207004_SM0002.pdf]

**Supplementary Movies**

***Movie 1***: http://www.youtube.com/watch?v=DqerNFysCBc&feature=plcp

***Movie 2***: http://www.youtube.com/watch?v=rtqsGLPaPSU&feature=plcp

***Movie 3***: http://www.youtube.com/watch?v=8PF8oKwUtxI&feature=plcp

***Movie 4***: http://www.youtube.com/watch?v=kCnU0pPbWB8&feature=plcp

***Movie 5***: http://www.youtube.com/watch?v=vD-iSc6eIX8&feature=plcp
